# Social Media as a Source of Knowledge about Gene Therapy for Spinal Muscular Atrophy

**DOI:** 10.3390/healthcare10122445

**Published:** 2022-12-04

**Authors:** Magdalena Tkaczuk, Dawid M. Zakrzewski, Maria Król, Marta Zawadzka, Przemysław M. Waszak, Maria Mazurkiewicz-Bełdzińska

**Affiliations:** 1 Department of Developmental Neurology, Medical University of Gdansk, 80-211 Gdańsk, Poland; 2 Department of Hygiene and Epidemiology, Medical University of Gdansk, 80-211 Gdańsk, Poland

**Keywords:** SMA, spinal muscular atrophy, onasemnogen abeparvovec, gene therapy, social media, Internet

## Abstract

Social media is one of the most common sources of medical information. We aimed to evaluate the information contained on websites, including social media and descriptions of fundraisers, in terms of the reliability of knowledge about SMA and gene therapy with onasemnogen abeparvovec. We used a set of available online links found using the Newspointtool. Initially, 1525 texts were included in the study, and after applying the inclusion and exclusion criteria, 112 texts were qualified for analysis using the DISCERN scale and the set of questions prepared by the authors. We observed that most of the texts had poor (48.65%) and medium (27.03%) reliability in the final reliability assessment. All the texts selected for the study were related to gene therapy, although few contained key information about it. In addition, the authors of the entries used various words and phrases that influenced the readers’ perceptions of the text. Of the analyzed sources, 68.8% had an emotional component. Social media is a poor source of information about gene therapy for SMA in Poland. The analyzed texts do not provide a full and complete description of the SMA problem. However, it is important to remember that the Internet is a changing source of information and will hopefully contain more relevant entries in the future.

## 1. Introduction

The Internet in the 21st century is the most popular source of information for an average person. In Poland, for more than half of the citizens, the Internet has become a main source of information [1]. It is the source most often used to search for answers to our most troublesome questions, including medical issues [2].The Internet has become more important than radio, television, and newspapers as sources of information. The main users of the Internet looking for health information are young people, mainly women, who suffer from chronic disease and visit doctors often. However, when looking for health-related information, the largest increase has been observed not in young but in older Internet users between 50 and 65 years old. In 2005, the percentage of men (52.1%) searching for health-related information was greater than the percentage of women (47.9%). However, in 2012, women (52.6%) were found to be the main searchers. Searching for health data on the Internet has increased by 25% over 7 years (2005–2012). [1] The most information searched on the Internet connected to medical issues is about health care and illnesses. Less often, people look for contact with healthcare workers or their own medical documentation [1].

However, is information found on the Internet reliable and factual? In this study, we analyzed articles, statements, and comments about the treatment of spinal muscular atrophy (SMA).

SMA is an autosomal recessive neuromuscular genetic disease [3,4]. After cystic fibrosis, SMA is the second-most-common autosomal recessive disease and can be lethal [3]. It is caused by amonozygotic pathogenic variant, mostly a deletion in the SMA1 gene [3,4].It is associated with the degeneration of alpha motoneurons, which results in progressive muscle weakness and atrophy. The proximal muscles are mainly involved. There are four degrees of severity. Depending on the subtype, symptoms may appear in infancy, making it completely impossible for those suffering from it to move [3].

Today, three treatments are available for SMA [4].The first and the only government-funded therapy in Poland until September 2022 is nusinersen therapy [5]. It is injected into the spinal canal every 4 months and can be applied to any SMA subtype, regardless of age and disease progression [6]. The second treatment option is risdiplam, which was available in Poland only in Early Access Programs until September 2022. Thereafter, data were available for patients with counter-indications for nusinersen. Risdiplam is a syrup taken daily and can be used from 2 months of age [4]. The third and the only one to which this article is devoted is gene therapy with onasemnogen abeparvovec. This involves intravenous injections of non-replicating adeno-associated virus capsids to deliver the proper SMA1 gene to motoneurons [4]. It raises hope among parents of children with SMA1, generating large amounts of fundraising on social networks to cover the approximate EUR 1.9 million purchase costs. This treatment in Poland was not covered by refund until September 2022. High expectations for this therapy, paid at enormous costs, are associated with emotional concern among parents and society. Hence, we decided to check the social media messages about this treatment.

The purpose of our work was to evaluate the information contained on websites, including social media, and descriptions of fundraisers in terms of the reliability of knowledge about SMA and gene therapy for the disease.

## 2. Materials and Methods

### 2.1. Search Strategy and Data Collection

A set of online-available texts was used in this study. All were found using Newspoint sp.zo.o. (Poland)—one of many commercially available Internet content monitoring tools. The software allowed us to extract data from portals, microblogs, videos, social media, and online forums. To identify any data about SMA with relevance to gene therapy, we used the search terms “SMA” OR “rdzeniowy zanik mięśni (spinal muscular atrophy)” AND“nusinersen” OR “spinraza” OR “gen*”. The “gen*” operator allowed us to use search terms, including “gen” as gene therapy, genetic therapy, zolgensma, and onasmenogen. Data export was carried out on 27 November 2021. Data were obtained for the period 27 November 2020 to 27 November 2021 (365 days). Every entry included the text, link, number of likes, follows, reach, and views. The initial database included 1525 links. The mentioned texts came from multiple social networking sites (Facebook and Twitter) and websites (journalistic and informational sites). The program analyzed and classified each piece of data into one of three categories: article (news text), comment (under an article or post), and statement (published statement as a new thread). All research data were publicly available, so no bioethics committee permission was necessary.

### 2.2. Inclusion and Exclusion Criteria

From the mentioned group, there were 1525 texts. The exclusion criteria included whether the data were duplicated on the same platform, hence excluding 1252 files. Numerous duplicates were related to multiple sharing of a given text by one platform while keeping the original source link and, thus, all statistics. A total of 273 files was retained for analysis. After analysis, 114 from 273 texts were included in the study, and inclusion criteria were mentioned for gene therapy. Two were excluded due to using a language other than Polish. Considering the above criteria, 112 articles were used in the study.

### 2.3. Scoring System

The collected texts were divided into three groups: articles (79), statements (19), and comments (14). They were then analyzed in terms of reliability and checked to determine whether they corresponded to current medical knowledge through questions, created by authors, concerning SMA symptoms, treatment, screening tests, rehabilitation, and diagnostics. Thereafter, the articles were assessed using the DISCERN (Quality Criteria for Consumer Health Information) scale. The DISCERN system is based at the University of Oxford, Division of Public Health and Primary Health Care, at the Institute of Health Sciences [7]. It is a validated instrument for assessing the reliability of medical information and is available online. It includes 16 questions about various key treatments contained in the publications, which are included in Table 1. For each question, the text was scored from 1 to 5 points (total sum from 16 to 80), whereas question 16 was a subjective assessment of the assessor of the credibility and reliability of a given text obtained after analyzing all the previous questions. If the source was a video platform, the video was viewed and treated as text, which was finally evaluated in DISCERN.

In this study, the total number of points obtained on the DISCERN scale was used for the final score of the reliability of articles. Points 16–26, 27–38, 39–50, 51–62, and 63–80 points indicated “very poor”, “poor”, “medium”, “good”, and “very good” levels of reliability, respectively [8].

### 2.4. Statistical Methods

Raw data were collected in Excel spreadsheets (Microsoft, Redmond, WA, USA). Statistical analysis was performed using STATISTICA 10.0 software (StatSoft Inc., Tulsa, OK, USA). All of the quantitative variables were tested using the Kolmogorov–Smirnov test to meet the criteria of a normal distribution (Gaussian distribution). Depending on whether the variables met the normality condition, appropriate statistical tests were applied at further stages. Continuous data were presented as medians and quartiles if they did not meet the conditions of the normal distribution. For comparisons between two groups, the parametric *t*-test or nonparametric Mann–Whitney U test were used. For the comparison of multiple groups, analysis of variance (for variables of parametric distribution) or the Kruskal–Wallis test (variables of non-parametric distribution) were used. For comparing qualitative survey data, Pearson’s chi-square test (with appropriate Yates’ Correction for small observed frequencies) was used. The cut-off level was set at *p* < 0.05 for statistical significance.

## 3. Results

### 3.1. Content of Included Data

In this study, 112 texts were used. The mentioned texts came from multiple social networking sites (Facebook and Twitter) and websites (journalistic and informational sites). The information relating to all of the sources used in the study is published in Table 2.

### 3.2. Quality Analysis

**Figure 1:**
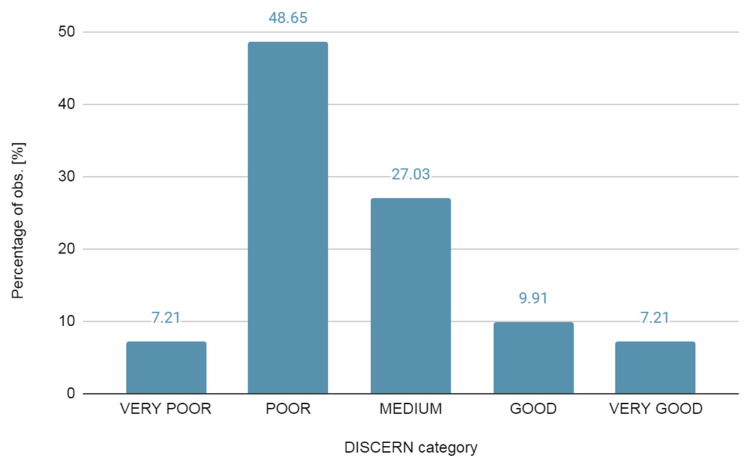
Percentage observed in DISCERN categories.

**Figure 2:**
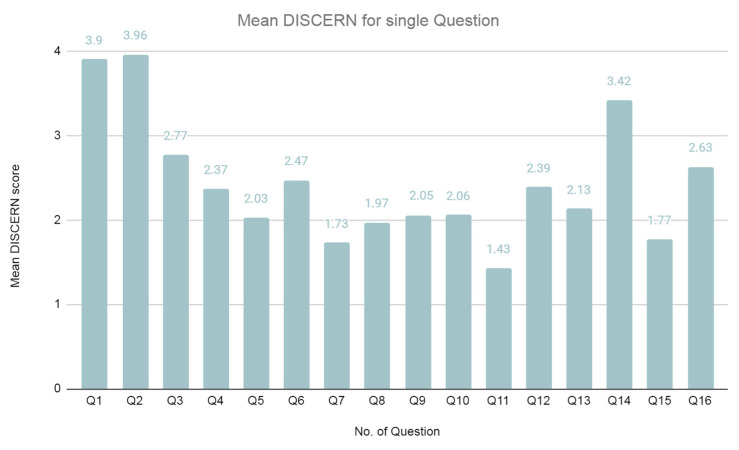
Mean DISCERN score for a single question.

We observed that most of the texts had poor (48.65%) and medium (27.03%) reliability levels in the final reliability assessment. The average DISCERN score was 39.66 points. The average DISCERN score for a single question was 2.18 points. The lowest mean score was obtained in question no. 11 (1.43 points) concerning the risk of the treatment used, followed by question no. 7 (1.73 points) concerning the inclusion of detailed sources of information in the text, and question no. 15 (1.74 points) concerning joint decision making on treatment. Questions 1 and 2 regarding the assumptions and goals of the texts in question scored the highest. Moreover, the highest score (63–80 points) on the DISCERN scale and the lowest score (16–26 points) were received by the same number of analyzed texts, namely, 7.20% each (Figure 1 and Figure 2).

### 3.3. Statements, Comments, and Articles

**Figure 3:**
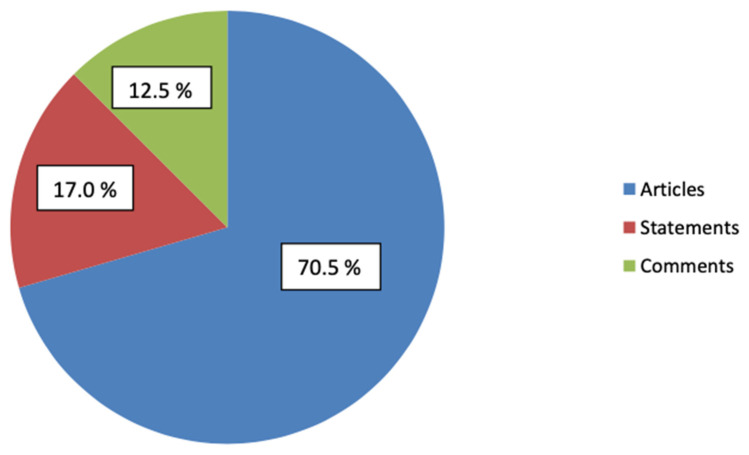
Division of the texts into subgroups.

All the texts were divided into three subgroups: comments (12.5%), statements (17%), and articles (70.5%) (Figure 3). The average number of points scored with DISCERN was 38.36, 39.15, and 40.05 points for statements, comments, and articles, respectively. The differences were not statistically significant. However, no correlation of the score obtained with DISCERN with the statistics on social media was found. In particular, statements in social media had greater indicators of reach, views, and shares than articles on other publishing scientific websites. Basic social media statistics of reaches and interactions are included in Table 3. Moreover, out of the three groups of data, statements from social media had the highest response rate in question 16, rated both the lowest (31.58%; 8.86% for articles and 15.38% for comments) and the highest (10.53%; 3.80% for articles and 7.69% for comments).

### 3.4. Relevant Information (Missing)

Although all the texts selected for the study were related to gene therapy, not all contained key information about it. Only 25.9% of the entries contained information about the intravenous dosing of this therapy, and 48.2% of the texts mentioned once-in-a-lifetime drug dosing. Moreover, 83.9% of the sources did not provide information on the safety of the drug supply, and 50.9% lacked information about the guidelines and limitations in supply (e.g., about a significant reduction in body weight, which cannot exceed 13.5 kg when taking the drug). Despite the little information contained in the data on gene therapy alone, 18.8% believed that it is 100% effective/always brings back all motor functions and can cure SMA. The action of the drug is determined individually and depends on the time of administration and the advancement of the disease, which excludes its 100% efficacy, and spreading false views on this subject can have enormous financial, psychological, and moral consequences, especially for parents of children affected by SMA.

### 3.5. Emotional Component

The patients’ parents often described their emotions and difficulties related to their children’s illness. In addition, the authors of the entries used words and phrases that influenced the readers’ perceptions of the text. Of the analyzed sources, 68.8% had an emotional component.

### 3.6. Alternative Treatment

Notably, at the time of data collection*, reimbursed drug therapy for children suffering from SMA was availablein Poland. The reimbursement for the drug nusinersen (Spinraza) was introduced in Poland on January 1, 2019. Analyzing the texts about gene therapy, we also verified the presence of information about this treatment method. In the analyzed sources, 26 (23.2%) of them did not contain any information about reimbursed treatment, and according to two (1.8%) of the sources, such treatment does not exist. Moreover, in as many as 35 (31.3%) of the texts, it is believed that treatment with nusinersen does not help (i.e., it does not bring any clinical improvement or stop the disease progression). In a separate question, the texts were also analyzed in terms of the expected results from nusinersen therapy. Furthermore, in 37 (33%) of the texts, the authors believed that nusinersen does not bring the expected results, which is more than 29 (25.9%) of texts mentioning the achievement of the expected results. Other drugs, such as risdiplam, were mentioned in 13.4% of the texts.

### 3.7. Description of the Disease

The main symptoms highlighted in the data were muscle weakness (63.7%) and motor developmental delay (44.6%). Correct intellectual development (2.7%) and lack of reflexes (8.9%) were very rarely mentioned. The form did not include questions about every possible symptom occurring in the SMA. Hence, there was a lack of information about tenuousness or other SMA-related disorders.

### 3.8. Diagnosis and Screening

In the vast majority of the data, the diagnostic process and which medical tests were performed were not specified. In the analyzed texts, the diagnosis process was mentioned only in 31.3% cases. In all of the texts, diagnosis of the disease was made based on a genetic test ordered by the specialist (31.3%). There is no self-diagnosis, no disease confirmation without genetic testing, and no genetic testing without a doctor’s prescription.

Among the collected materials, 69.6% contained no information about SMA screening in newborns, and 8.9% of such screenings in Poland did not exist. This may be due to the process of initiating screening in subsequent Polish provinces toward SMA in the period from which the above data were derived.

### 3.9. Rehabilitation

The issue relating to the need for rehabilitation appeared in less than half of the sources (48.2%), whereas the need for fundraising for rehabilitation and information about its reimbursement was completely ignored in the vast majority of the analyzed sources.

The set of questions contained in the formula, with the percentages of answers, can be found in Table 4.

## 4. Discussion

### 4.1. Current Study

When creating the study proposal, we asked ourselves one key question: Is the information found on the Internet reliable and factually correct? Given the media hype surrounding the expensive SMA gene therapy, we knew that the answer was not going to be simple. We observed that most of the texts had a poor (48.65%) reliability level, but the mean DISCERN score was 39.66 points, which indicates that the online material on the gene therapy is of “medium” quality and leaves recipients with an incomplete, but not regrettable, picture of the disease. The low quality of the above information also confirmed a low (2.18 points) average DISCERN score for a single question. Moreover, there was much missing relevant information about the dosing, supply, safety, guidelines, and limitations of gene therapy and the symptoms, screening tests, or refund treatments, which are essential for a full understanding of the disease. Furthermore, many of the entries were fundraising-related texts. It is understandable that if parents want to collect money for the treatment, it is not the most important case to educate precisely about the side effects, doses, or other available therapies in Poland. Combined with the strong emotional content of the texts, this can be perceived as a deliberate effort by the authors, which made the disease seem more terrible and mysterious. In this way, they can try to arouse greater emotions in the recipients and encourage them to donate funds to the fundraiser for treatment.

The texts were separately analyzed as statements, comments, and articles. Is there a difference between the subgroups? All the results were not statistically significant, but statements had the highest response rate in question 16, rated both the lowest and the highest. This may indicate a growing interest in SMA and awareness and treatment of the disease. This interest has been growing in recent years in an attempt to reduce the limited access to information often superficially disseminated by collection websites. Hence, we can now see both texts with very poor and very good levels of quality.

### 4.2. The Internet as a Source of Knowledge in Other Works

Social media and the Internet are widely researched sources of information on medical topics. Unfortunately, it has been shown that 40% of the most common shared medical-related links in Polish language social media contain false information [9].

One of the most visited websites with videos in search of knowledge is YouTube [10], which, according to researchers, is not very reliable [11].

Krakowiak et al. [12] checked whether information on meningiomas on the Internet is qualitative. Based on the DISCERN form, it was concluded that the information contained in the videos on meningiomas presented on YouTube is of poor quality. The study showed that 47.9% were rated “poor”, whereas only 9.24% were rated as “good”. Comparing it to the results obtained in our work, where the grade “poor” was given to 48.65%, and the grade “good” only to 9.91%, we can notice that the Internet and social networking sites are sources of low-quality information.

A different conclusion was found by various researchers. Szmuda et al. [13] checked the reliability of the information from the first 30 movies displayed for entries related to cerebral ischemia. Again, in this work, the DISCERN scale was used to assess reliability, but the results differed from the previous ones. Most of these videos were published by doctors, hospitals, or other medical institutions. Therefore, the quality of the information provided was high. This difference in results with our study may come from the frequency of occurrence of a given disease entity. Stroke, affecting a large part of the population, was more likely to be the subject of choice by specialists, because the knowledge imparted can help more patients. At the same time, there are more entries about it, and the best-quality videos were the most viewed, ranking in the highestplaces. In our study, we did not limit ourselves to the proposals selected for us by the algorithm but analyzed all available entries.

Arikanoglu et al. [14] assessed the usefulness of the information contained in 100 films displayed for the slogan “restless leg syndrome” using the subjective scale global quality scale. As many as 77% of the films were considered useful because they contained reliable information about any issue related to restless leg syndrome (RLS). The authors of the work, however, had an important observation—even videos in the useful category did not provide a full and complete description of the RLS. This connects their conclusions with our work, alerting them that the information contained in social media is often incomplete and thus distorts the reception of the whole issue.

Our study aimed to check the reliability of Internet knowledge about SMA on a broader plane, and sources from more than one specific portal were used for the analysis, similar to the reports of Iyer et al., Slick et al., and Ahmed et al.

Iyer et al. [15], similar to the current study, conducted a study related to the SMA information contained in the network. A group of 20 patients or their families with SMA was examined. They were given a detailed interview to determine how information on social media is used to obtain information about treatment with nusinersen. As in our analysis, the weaknesses of using social media were the lack of reliability of the information provided there and the violation of privacy when participating in Facebook support groups. This was understandable because, as we saw in our database, there was more information with an emotional component than with reliable medical knowledge. Moreover, misinformation or false information can cause a conflict in the doctor–patient relationship. This can make the diagnostic and treatment processes difficult. On the positive side, emotional support was provided in groups on Facebook, as well as detailed descriptions of procedures, photos, and videos from patients or their parents who had undergone the drug administration procedure. According to the opinions of the respondents, such support groups are not widely available outside of Facebook, which the authors of these works noticed as well, during the assessment. A similar analysis was carried out in the study on sickle cell disease (SCD) by Slick et al. [16], who checked the accuracy of information on SCD in social media. They showed that 55.1% of medical posts contain incorrect or imprecise information about the disease, and 44.9% of the posts contain correct data. The high percentage of accurate data from this study, which partially differs from those obtained in our study, may be related to the disease entity or the popularity of its occurrence.

Given that the Internet is such a wide source of information, it is important to consider how it affects the treatment process today. Ahmed et al. [17] compared the perspectives of patients and doctors while searching social media for information on irritable bowel disease. In this work, as in ours, we showed that knowledge contained on the Internet often comes from unreliable and untested sources. When considering biological medications, 55% described only the negative aspects of this treatment, and 38% described only its positive aspects. The treatment presented in this way makes it difficult to form an objective opinion about it. This diversity is similar to our analysis of current refund treatment with nusinersen. In only 58% of texts do people believe that nusinersen helps, and only 26% are satisfied with its results. In addition, as in the above-mentioned study, the aspects of the threat to privacy, personal data leakage, and further consequences of such a platform were raised. The positive aspects of using the Internet have also been noticed, such as the presence of webinars for patients, conducted by specialists, emotional support among users of social media, and ease of sharing information published by specialists. Moreover, what is an advantage for both the doctor and the patient is the possibility of consultation of test results, treatment results, and new forms of therapy between specialists from around the world.

Unfortunately, disinformation on the Internet is not a new phenomenon that only affects patients with SMA. We can read about any number of diseases on the Internet. Desai et al. [18] listed social media tools used to spread false information. It is important for individuals to be able to discern valid sources of scientific information on the Internet, as many sites freely and knowingly spread false information. It should be remembered that social media functions based on algorithms, which means that people with specific views are often contacted and grouped with people with the same beliefs. This makes it difficult to form an objective opinion. There are also bots on the Internet (only 53% of Twitter users are people) who intentionally spread false information. It is not unusual, then, that so much of the information on SMA that we examined in our work was of poor quality.

In the listed articles, researchers came to different conclusions about the quality of information shared on social media. Their wide range consisted of many factors, including the frequency of the disease, the source of the entry, and the authors’ assumptions and goals. It is important for physicians/health professionals to be aware of what information is appearing on social networking sites and which of them are deliberately or not omitted. This will make it easier to contact the parents of children with SMA and contribute to drawing attention to aspects that are not readily available on the Internet.

We hope that the quality and reliability of the content scientists publish online will improve over time.

### 4.3. Limitations and Advantages

The limitations of the article are caused by the dynamics of changes taking place on the Internet. The survey is presented as a snapshot of what was present on Internet portals at a given moment. Therefore, it is difficult for the study to reflect on the constant changes and new data.

It should be emphasized that texts related to gene therapytreatment were included in the study, so the authors cannot be accused of omitting information on the description of the disease, diagnosis, or rehabilitation. However, considering how important and costly this treatment is, we believe that it is essential to present the full issue and quality of care for patients with SMA in Poland and not to be limited to incomplete pieces of information that are often insufficient and perhaps even untrue.

As we can imagine, some of the comments were just short, few-sentence texts. It was difficult to evaluate such a short inscription in every question on the DISCERN scale. Moreover, many of the entries did not have the purpose of educating readers in the most precise way. Many of them were fundraising-related texts, where the most essential goal was to collect money for gene therapy. There were only a few educational entries in the entire study.

The advantage of this article is that information from a wide variety of databases was considered. Many entries from various websites and social networking sites were analyzed. This ensures the uniformity of the data provided and increases the reliability of the results obtained. The DISCERN scale, which we used to classify the entries, was rated by more than one researcher, making it more impartial.

### 4.4. Future Directions

Our analysis only included Polish texts. Hence, the quality of knowledge in other countries may be different, as can a summary point of view of this topic. In future studies, the authors can focus on the subject in a wider range. We believe that checking the quality of data on the Internet in English can be the first move to expand this study.

Moreover, this article was written before the refund of the onasemnogen abeparvovec gene therapy in Poland, which began on 1 September 2022, and will most likely change the distribution of online information on the entire issue of SMA. We hope that this will be a turning point to increase the reliability of the published materials on treatment with this therapy, which should be verified in future studies.

## 5. Conclusions

The Internet is a source of a wealth of information on SMA. The information provided in it reaches a wide audience.

Most of the entries appearing in our study were of low quality, with poor (48.65%) or medium (27.03%) reliability levels in the final assessment. Furthermore, most of them do not cover the entire topic of SMA or key information on gene therapy.

Out of all the sources, 83.9% did not provide information on the safety of the drug supply, and 50.9% did not contain information about the guidelines and limitations in supply. Furthermore, 68.8% had an emotional component that could influence readers’ perceptions of the texts. The results confirm that the Internet is not always a source of reliable information.

These results are part of the ongoing discussion about the reliability of medical knowledge on the Internet. Our study, however, argues for the low credibility of information found in social media.

Future research should focus on a comparison of whether onasemnogen abeparvovec reimbursement will affect the quality and number of appearing entries on gene therapy in Poland and whether there are new concerns and problems related to gene therapy.

## Figures and Tables

**Table 1:** DISCERN instrument variables.

No. of Question	The Question
1	Are the aims clear?
2	Does it achieve its aims?
3	Is it relevant?
4	Is it clear what sources of information were used to compile the publication (other than the author or producer)?
5	Is it clear when the information used or reported in the publication was produced?
6	Is it balanced and unbiased?
7	Does it provide details of additional sources of support and information?
8	Does it refer to areas of uncertainty?
9	Does it describe how each treatment works?
10	Does it describe the benefits of each treatment?
11	Does it describe the risks of each treatment?
12	Does it describe what would happen if no treatment is used?
13	Does it describe how the treatment choices affect overall quality of life?
14	Is it clear that there may be more than one possible treatment choice?
15	Does it provide support for shared decision-making?
16	Based on the answers to all of the above questions, rate the overall quality of the publication as a source of information about treatment choices

Source: http://www.discern.org.uk/discern_instrument.php, accessed on 1 March 2022.

**Table 2:** Most common data sources included in the study.

	Initially Found (Not Duplicated)	Finally Used	% of Total
**Facebook**	87	24	21.05%
**Nasze Miasto**	10	6	5.26%
**Wykop**	12	6	5.26%
**Bomega**	6	4	3.51%
**Termedia**	9	4	3.51%
**TwojeMiasto**	5	4	3.51%
**kielce.tvn.pl**	3	3	2.63%
**lubelskie.pl**	3	3	2.63%
**YouTube**	7	3	2.63%
**24 kurier**	1	2	1.75%
**comments.disqus.com**	2	2	1.75%
**dziendobry.tvn.pl**	2	2	1.75%
**Dziennik Wschodni**	2	2	1.75%
**Fakty Kaliskie**	2	2	1.75%
**Gazeta Krakowska**	2	2	1.75%
**Interia**	4	2	1.75%
**kobietach.pl**	2	2	1.75%
**medexpress.pl**	2	2	1.75%
**parenting.pl**	2	2	1.75%
**RMF24.pl**	2	2	1.75%
**Twitter**	17	2	1.75%

The other texts were a fraction of a percent.

**Table 3:** Basic social media statistics of the included material.

	Mean Views	SD	Mean Reach	SD	Mean Likes	SD	Mean Followers	SD
Articles	1.5 *	12.3	25,761.8	55,698.8	73.4 **	524.1	17,524.6 **	131,159.2
Comments	25.8 *	47.7	354.7	765.1	3.6	8.5	0.0	0.0
Statements	61.7 *	53.1	65,171.4	215,937.7	1086.4 **	3102.4	115,412.5 **	284,345.7
Total	10.9	31.8	29,601.1	101,443.5	234.6	1372.6	31,940.0	163,306.0

* *p* < 0.01, ** *p* < 0.05.

**Table 4:** Information and statements found in the analyzed material.

	Count	Cumulative	Percent	Cumulative
Category	Is there a treatment reimbursed in Poland for SMA?			
Yes	84	84	75.00000	75.0000
No	2	86	1.78571	76.7857
Missing	26	112	23.21429	100.0000
Category	Does treatment with Nusinersen help the patients?			
Yes	65	65	58.03571	58.0357
No	12	77	10.71429	68.7500
Missing	35	112	31.25000	100.0000
Category	Does Nusinersen cause serious side effects?			
Yes	1	1	0.89286	0.8929
No	6	7	5.35714	6.2500
Just pain	5	12	4.46428	10.7143
Missing	100	112	89.28571	100.0000
Category	Does Nusinersen therapy bring the expected results?			
Yes	29	29	25.89286	25.8929
No	37	66	33.03571	58.9286
Missing	46	112	41.07143	100.0000
Category	Does Zolgensma helps in 100% of cases?			
Yes	32	32	28.57143	28.5714
No	21	53	18.75000	47.3214
Missing	59	112	52.67857	100.0000
Category	Are there guidelines for the supply of the drug, e.g., body weight?			
Yes	55	55	49.10714	49.1071
No	20	75	17.85714	66.9643
Missing	37	112	33.03571	100.0000
Category	Is newborn screening for SMA carried out in Poland?			
No	10	10	8.92857	8.9286
Yes	24	34	21.42857	30.3571
Missing	78	112	69.64286	100.0000
Category	Does the text contain emotional components?			
No	34	34	30.35714	30.3571
Yes	77	111	68.75000	99.1071
Missing	1	112	0.89286	100.0000
Category	Are other drugs used in SMA?			
No	3	3	2.67857	2.6786
Yes	15	18	13.39286	16.0714
Missing	94	112	83.92857	100.0000
Category	Is the health system in SMA therapy efficient?			
Yes	27	27	24.10714	24.1071
No	11	38	9.82143	33.9286
Missing	74	112	66.07143	100.0000
Category	Is gene therapy given intravenously?			
Yes	29	29	25.89286	25.8929
No	2	31	1.78571	27.6786
Missing	81	112	72.32143	100.0000
Category	Is gene therapy administered once?			
No	1	1	0.89286	0.8929
Yes	54	55	48.21429	49.1071
Missing	57	112	50.89286	100.0000
Category	Is it safe to give gene therapy?			
No	10	10	8.92857	8.9286
Yes	4	14	3.57143	12.5000
Not always	4	18	3.57143	16.0714
Missing	94	112	83.92857	100.0000
Category	Is muscle weakness a symptom of SMA?			
Yes	71	71	63.39286	63.3929
Missing	41	112	36.60714	100.0000
Category	Is motor developmental delay a symptom of SMA?			
Yes	50	50	44.64286	44.6429
No	3	53	2.67857	47.3214
Missing	59	112	52.67857	100.0000
Category	Is intellectual development normal in the course of SMA?			
No	2	2	1.78571	1.7857
Yes	3	5	2.67857	4.4643
Missing	107	112	95.53571	100.0000
Category	Is there a noticeable absence of reflexes in the course of SMA?			
Yes	10	10	8.92857	8.9286
Missing	102	112	91.07143	100.0000
Category	Was the genetic test done after consulting a doctor?			
Yes	35	35	31.25000	31.2500
Missing	77	112	68.75000	100.0000
Category	Was the genetic test done without consulting a doctor?			
No	26	26	23.21429	23.2143
Missing	86	112	76.78571	100.0000
Category	Didthe doctor diagnose the disease without genetic testing?			
No	25	25	22.32143	22.3214
Missing	87	112	77.67857	100.0000
Category	Has the disease been diagnosed after autodiagnosis?			
No	26	26	23.21429	23.2143
Missing	86	112	76.78571	100.0000
Category	Is rehabilitation in SMA necessary?			
Yes	54	54	48.21429	48.2143
Missing	58	112	51.78571	100.0000
Category	Is rehabilitation reimbursed?			
No	5	5	4.46429	4.4643
Yes	1	6	0.89286	5.3571
Missing	106	112	94.64286	100.0000

## Data Availability

The data that support the findings of this study are openly available via the Internet (see Methods Section). Detailed databases used in this research are available from the corresponding author upon reasonable request.
